# Molecular characteristics of group B Streptococcus isolates from infants in southern mainland China

**DOI:** 10.1186/s12879-019-4434-0

**Published:** 2019-09-18

**Authors:** Juan Li, Wenjing Ji, Kankan Gao, Haijian Zhou, Lihua Zhang, Xiaoping Mu, Chunlei Yuan, Xiaoshan Guan, Qiulian Deng, Lian Zhang, Huamin Zhong, Xiurong Gao, Fei Gao, Yan Long, Chien-Yi Chang, David J. McIver, Haiying Liu

**Affiliations:** 1 Clinical Laboratory, Guangzhou Women and Children’s Medical Center, Guangzhou Medical University, Guangzhou, 510623 Guangdong China; 2 Clinical Laboratory, Affiliated Cancer Hospital & Institute of Guangzhou Medical University, Guangzhou, 510095 Guangdong China; 3 Department of Pharmacy Administration and Clinical Pharmacy, School of Pharmacy, Xi’an Jiaotong University, Xi’an, 710049 Shaanxi China; 4 Center for Drug Safety and Policy Research, Xi’an Jiaotong University, Xi’an, 710049 Shaanxi China; 5 National Institute for Communicable Disease Control and Prevention, and State Key Laboratory for Infectious Disease Prevention and Control, Chinese Center for Disease Control and Prevention, Beijing, 102206 China; 6 Clinical Laboratory, Dongguan Tungwah Hospital, Sun Yat-Sen Universtiy, Dongguan, 523110 Guangdong China; 7 Clinical Laboratory, Guangdong Women and Children’s Hospital, Guangzhou, 511400 Guangdong China; 8 Clinical Laboratory, ZhongshanBoai Hospital, Zhongshan, 528403 Guangdong China; 9 Department of Neonatalogy, Guangzhou Women and Children’s Medical Center, Guangzhou Medical University, Guangzhou, Guangdong China; 10 School of Chemistry and Biosceinces, University of Bradford, Bradford, UK; 11 Metabiota Inc, Nanaimo, Canada

**Keywords:** Group B *Streptococcus*, Infant, Molecular characterization, Sequence types, CC17, *hvgA*

## Abstract

**Background:**

Invasive group B *Streptococcus* (GBS) disease in Chinese infants has gradually gained attention in recent years, but the molecular epidemiology of the pathogen is still not well known.

**Methods:**

This multicenter study retrospectively investigated distribution of capsular serotypes, sequence types (STs), and hypervirulent GBS adhesin gene (*hvgA*) in clinical GBS isolates that caused invasive disease in infants aged < 3 months of age in southern mainland China between January 2013 and June 2016. Genes for antibiotic resistance to tetracycline, erythromycin, and clindamycin were also examined.

**Results:**

From a total of 93 GBS isolates taken from 34 early-onset disease (EOD, 0–6 days after birth) and 59 late-onset disease (LOD, 7–89 days after birth) cases, four serotypes were identified: serotypes III (79.6%), Ib (12.9%), Ia (4.3%), and V (3.2%). Serotype III accounted for 73.5% of EOD and 83.1% of LOD and was responsible for 75.5% of cases involving meningitis. Fifteen STs were found, with the majority being ST17 (61.3%), ST12 (7.5%), ST19 (7.5%), and others (23.7%). 96.8% of STs belonged to only five clonal complexes (CCs): CC17 (64.5%), CC10 (12.9%), CC19 (9.7%), CC23 (6.5%), and CC1 (3.2%). The *hvgA* gene was detected in 66.7% of GBS isolates and 95% of CC17 isolates, all of which were serotype III except one serotype Ib/CC17 isolate. A large proportion of GBS isolates were found to be resistant to tetracycline (93.5%), clindamycin (65.5%), and erythromycin (60.2%). Genes of *tetO* (74.7%) and *tetM* (46.0%) were found in tetracycline resistant isolates, *linB* (24.6%) in clindamycin resistant isolates, and *ermB* (87.5%) and *mefA* (3.6%) in erythromycin resistant isolates.

**Conclusion:**

Our results reveal higher prevalence of serotype III, ST17, CC17, *hvgA* expressing, and antibiotic resistant GBS isolates than previously reported in southern mainland China.

This study provides guidance for appropriate measures of prevention and control to be taken in the future.

## Background

Group B *Streptococcus* (GBS), also called *Streptococcus agalactiae*, is a main causative pathogen of invasive neonatal bacterial infection, causing high morbidity and mortality [1–3]. A meta-analysis by Madrid et al. found that the incidence of invasive GBS disease in infants worldwide was 0.49 per 1000 live births (95% confidence interval [CI], 0.43–0.56), and was highest in Africa (1.12) and lowest in Asia (0.30) [4]. Some potential factors which may contribute to the apparent lower incidence in Asia include the low overall prevalence of maternal colonization, incomplete case ascertainment, and the lack of well-trained clinicians and appropriate laboratory facilities to identify the disease, especially in primary healthcare centers [5]. In this meta-analysis, early-onset disease (EOD) and late-onset disease (LOD) were defined as invasive GBS infections occurring 0–6 days and 7–89 days after birth, respectively [6]. GBS is a highly diverse organism and commonly colonizes the human gastrointestinal and genitourinary tracts. Based on capsular polysaccharides (CPS), GBS can be classified into 10 serotypes including Ia, Ib, and II through IX. However, only limited subtypes have been identified in infants with invasive infection, indicating that variation in expressed surface molecules is related to pathogenicity of strains. The Madrid et al. meta-analysis found that serotype III accounted for the highest proportion of serotypes globally (61.5%) [4], while our group’s recent study found the proportion of serotype III in Guangdong province was 77.9% [7], much higher than the global level. Jones et al. analyzed GBS isolates with multilocus sequence typing (MLST) and reported that serotype III strains have been categorized to ST17 and belong to complex clone 17 (CC17) [8]. In the CC17 lineage, which is strongly associated with neonatal meningitis, the *hvgA* gene encoding a surface-anchored protein named hypervirulent GBS adhesin (HvgA) has been identified [9]. The HvgA surface-anchored protein not only acts as an important adhesion medium for destroying and penetrating the intestinal and blood-brain barriers, but also mediates the migration of bacteria into the bloodstream and the central nervous system, which leads to infection [9, 10].

In addition to expressing many factors which help it evade immune detection and clearance, acquiring antimicrobial resistance genes is another strategy observed in GBS to improve survival within the host. Penicillin G and ampicillin are recommended by U.S. CDC as the first-line drugs for prophylaxis and treatment of neonatal GBS infection, while clindamycin was the recommended alternative for individuals with a β-lactam allergy [6, 11]. Unfortunately, the proportion of isolates reported to be resistant to erythromycin and clindamycin has increased in recent years, and especially high macrolide resistance in GBS have been detected among isolates circulating in both infected infants and adult female carriers in China [11–13].

Although invasive GBS infection in Chinese infants has gradually gained attention in recent years, no standardized or official guidelines for GBS prevention and control exists and the molecular epidemiology of the pathogen remains not well known. Furthermore, published studies have shown that geographical and ethnic differences in hosts effect the molecular epidemiology of invasive GBS [9, 14–17]. By determining serotypes, sequence types (STs), *hvgA* expression and antibiotic resistance of GBS isolates from infants with invasive infections in four hospitals in Guangdong province, China between January 1, 2013 and June 30, 2016, this study aims to elucidate the molecular characteristics of neonatal GBS disease prevailing in southern mainland China. This work will be helpful for exploring preventive and treatment strategies for GBS infections in southern mainland China and for the development of serotype-based vaccines in the future.

## Methods

### Study design

The study population was comprised of infants younger than 90 days of age with GBS isolated from a normally sterile medium including blood, cerebrospinal fluid (CSF), synovial fluid, and/or bone marrow, from the following four hospitals in Guangdong, China, between January 1, 2013 and June 30, 2016: Guangzhou Women and Children’s Medical Center, Dongguan Tungwah Hospital, Guangdong Women and Children’s Hospital, and Zhongshan Boai Hospital. We retrospectively collected demographic information, clinical signs and symptoms, and laboratory data for each GBS patient from Hospital Information Systems (HIS) and medical records at each study site.

GBS sepsis was defined as neonates in whom GBS was cultured from blood with clinical symptoms or signs including fever, respiratory distress, apnea, cyanosis, poor feeding, jaundice, lethargy, and seizure. Additionally, meningitis was diagnosed if a) CSF was cultured positive for GBS, or b) GBS negative but with a cellular reaction of more than 20 leukocytes/μL in CSF associated with GBS positive blood culture and consistent clinical manifestation.

### GBS isolates

All GBS strains were confirmed by the four study hospitals to be *S. agalactiae* using VITEK 2 COMPACT microbiology systems (BioMerieux, Marcy L’Etoile, France). GBS isolates collected during the study period were centralized in the microbiology laboratory of Guangzhou Women and Children’s Medical Center for analysis, where they were cultured at 37 °C in 5% CO_2_ in trypticase soy agar supplemented with 5% sheep blood. We used ATCC 2592 and ATCC 49619 as quality control bacteria.

### Capsular serotyping

GBS serotyping was performed using Strep-B-Latex® rapid latex agglutination test kit (Statens Serum Institute, Hillerød, Denmark), according to the manufacturer’s instructions. The capsular genotype was verified using a previously described multiplex PCR assay [18].

### Multilocus sequence typing (MLST)

Genomic DNA was prepared from overnight GBS cultures by a standard protocol for Gram-Positive bacteria, using Bacterial Genomic DNA kit (Takara Code NO.9164, Japan). Typing was performed by sequencing the internal fragments of seven house-keeping genes (*adhP, pheS, atr, glnA, sdhA, glcK,* and *tkt*). PCR was used to amplify genomic DNA using the primer pairs indicated at http://pubmlst.org/sagalactiae/info/primers.shtml. PCR products were purified and sequenced in both directions by BGI Tech Solutions Co. Ltd. Alleles and sequence types (STs) were determined using the *S. agalactiae* MLST website (http://pubmlst.org/sagalactiae/). Sequence types that shared five or more alleles of the seven loci were clustered into a clonal complex (CC) using eBURST software. The term “singleton ST” refers to an ST that did not cluster into a CC. BioNumerics software version 5.1 (Applied Maths, Belgium) was used to create minimum spanning trees to illustrate relationships between MLST and CCs.

### Detection of hypervirulent GBS adhesion (*hvgA*) gene

The *hvgA* gene was amplified by PCR using previously described primers ST17S and ST17AS [18]. Amplification products were purified and sequenced by BGI Tech Solutions Co. Ltd.

### Antibiotic resistance

We used VITEK 2 COMPACT microbiology systems to determine the minimum inhibitory concentrations (MICs) of the following 11 antibiotics: penicillin, ampicillin, ceftriaxone, vancomycin, linezolid, erythromycin, azithromycin, levofloxacin, ofloxacin, ciprofloxacin and clindamycin. The criteria of the Clinical and Laboratory Standards Institute (CLSI), 2016 edition, were applied for interpretation. *S. pneumonia* ATCC 49619 and *Staphylococcus aureus* ATCC25923 were used as quality control strains in each set of tests to ensure accuracy of results. Erythromycin resistant genes *erm(A), erm(B), mef(A),* and tetracycline resistant genes *tet(L), tet(K), tet(O), tet(m)*, being the most common antimicrobial genes, were detected by PCR. The primers and PCR conditions followed previously described methods [19, 20].

### Statistical analysis

Continuous data was expressed in terms of numbers and percentages and Fisher’s exact test was used for comparison of categorical variables. Statistical analyses were performed using SPSS software (ver. 21.0; SPSS Inc., U.S). A *p*-value of < 0.05 was considered statistically significant. All antibiotic susceptibility data were extracted using WHONET 5.6 software, as recommended by the World Health Organization (WHO).

## Results

A total of 93 non-duplicate GBS isolates which caused invasive disease in infants were recovered; among them were 65 from Guangzhou Women and Children’s Medical Center, 12 from Dongguan Tungwah Hospital, 8 from Guangdong Women and Children’s Hospital and Health Institute, and 8 from Zhongshan Boai Hospital. GBS identification by the Vitken Compact 2 system were confirmed for all isolates using mass spectrometry. A total of 34 cases (36.6%) were diagnosed as EOD and 59 (63.4%) were diagnosed as LOD (Table 1). There were 49 GBS cases which were diagnosed with meningitis, 47% (16/34) of which were observed in EOD cases and 55.9% (33/59) in LOD cases. Overall, there were 43 GBS cases which were diagnosed with sepsis, and one case with arthritis.

**Table 1:** Clinical information of GBS strains by isolate source and disease onset

Culture source	Early-onset disease (*n* = 34)	Late-onset disease (*n* = 59)	Total (*n* = 93)
Blood, n (%)	28 (82.4)	34 (57.6)	62 (66.7)
Cerebrospinal fluid, n (%)	1 (2.9)	16 (27.1)	17 (18.3)
Cerebrospinal fluid+blood, n (%)	5 (14.7)	8 (13.6)	13 (14.0)
Articular cavity fluid, n (%)	0	1 (1.7)	1 (1.1)
Bacteremia, n (%)	18 (53.0)	25 (42.4)	43(46.2)
Meningitis, n (%)	16 (47.0)	33 (55.9)	49(52.7)
Septic arthritis, n (%)	0	1 (1.7)	1(1.1)

### Serotype distribution

**Fig. 1:**
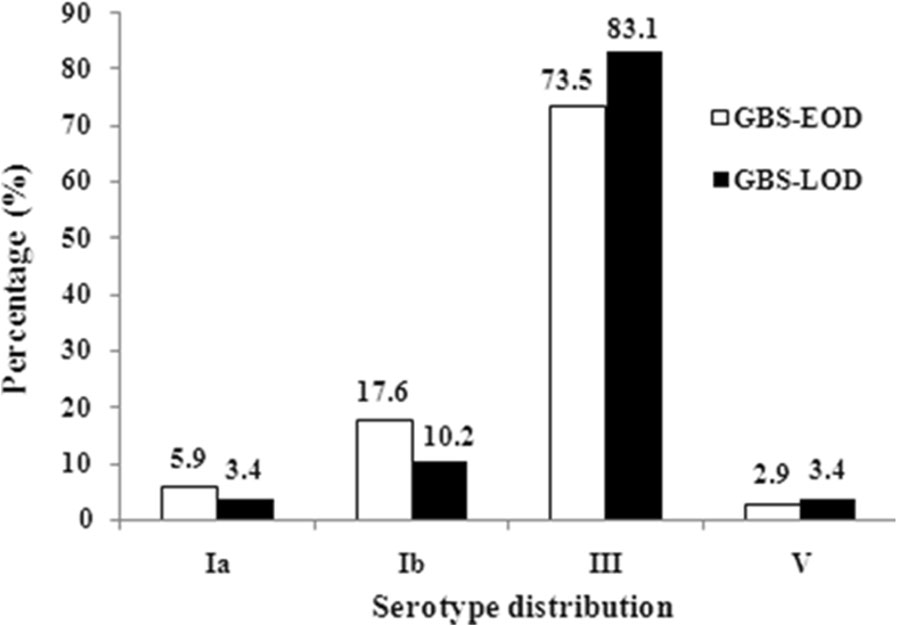
Serotype distribution of GBS isolates from early-onset disease and late-onset disease cases of GBS. The percentage of serotype III was the highest among four serotypes, both in EOD and LOD. GBS, group B streptococcus; EOD, early-onset disease; LOD, late-onset disease

Four capsular types were identified among the 93 isolates. Serotype III was the most prevalent (74, 79.6%), followed by Ib (12, 12.9%), Ia (4, 4.3%) and V (3, 3.2%). Serotype III was also the most prevalent serotype of both EOD (73.5%, 25/34) and LOD (83.1%, 49 /59) cases (Fig. 1). Among the 49 cases with meningitis, serotype III accounted for 62.5% (10/16) and 81.8% (27/33) in EOD and LOD cases, respectively. There was no difference in distribution of serotype III between the meningitis group (37/49) and non-meningitis group (37/44, *P* = 0.440).

### Multilocus sequence typing

MLST analysis revealed the presence of 15 separate STs among the 93 invasive GBS isolates. The relationships between the STs, CCs, and serotypes of the 93 invasive GBS strains are presented in Table 2. The most frequent ST was ST17 (57, 61.3%), followed by ST12 (7, 7.5%), ST19 (7, 7.5%), and ST23 (4, 4.3%).

**Table 2:** Distribution of sequence types and *hvgA* gene in GBS isolates across serotype

Serotype (n)	CC (ST, n)	CC17 (n)	*hvgA+* (n)
Ia (4)	CC23 (ST23, 3; ST24, 1)	–	–
Ib (12)	CC10 (ST10, 1; ST12, 7; ST579, 1)	–	–
	CC17 (ST357, 2)	2	1
	CC23 (ST23, 1)	–	–
III (74)	CC17 (ST17, 57; ST188, 1)	58	56
	CC19 (ST19, 7; ST27, 2)	–	1
	CC10 (ST562, 1; ST399, 2)	–	3
	CC23 (ST55, 1)	–	1
	ST651 (3)^a^	–	–
V (3)	CC1 (ST1, 3)	–	–
Total (93)		60 (64.5%)	62 (66.7%)

*CC* clonal complex, *ST* sequence type, *hvgA* hypervirulent GBS adhesion gene

^a^ST651 was a singleton

**Fig. 2:**
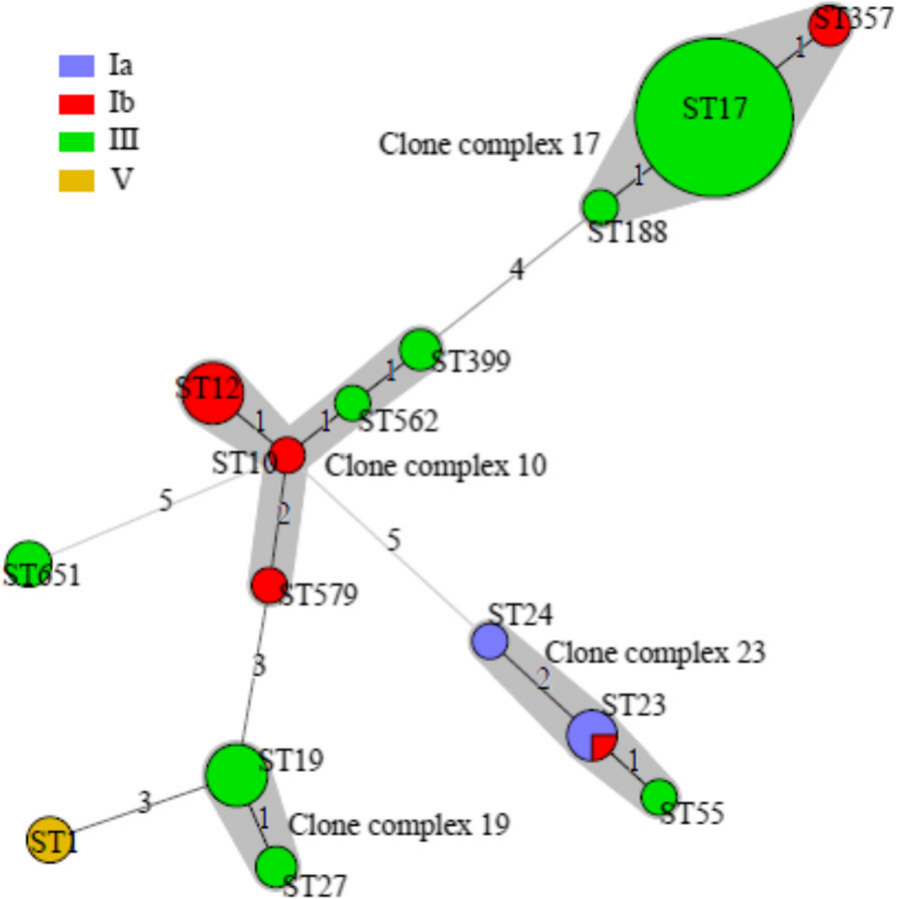
Correlation between clone complexes (CCs), sequence type (ST) and serotypes of invasive group B Streptococcus isolates. In the minimum spanning tree, the STs are displayed as circles. The size of each circle indicates the number of isolates of that particular ST, with each colour represents a different serotype. The founder ST was defined as the ST with the greatest number of single-locus variants. The major clonal complexes (CCs) are indicated in the diagram. The colours of the halo surrounding the STs denote types that belong to the same CC. STs that vary by one allele in their multilocus sequence typing profiles (single locus variants) are arranged in circles around the primary founder sequence type. The numbers on the lines represent the number of locus variants. ST17 was the most frequent ST among 15 STs identified. Most of GBS isolates were found within five clonal complexes (CCs): CC17, CC10, CC19, CC23, and CC1, with CC17 being the most common

Fourteen of the STs were clustered into five CCs (1, 10, 17, 19, and 23), and one ST651 was a singleton. Among the 93 isolates, 90 (96.8%) were found within five CCs: CC17 (64.5%), CC10 (12.9%), CC19 (9.7%), CC23 (6.5%), and CC1 (3.2%). Of all isolates, 3 (3.2%) were identified as singleton ST651 (Fig. 2). The serotype and MLST analyses showed that isolates of the same CC usually expressed one dominant serotype, indicating a relationship between CCs and serotypes. Of the 74 serotype III strains, 2 major CCs were detected, where 58 (78.4%) strains belonged to CC17 and 9 (12.2%) belonged to CC19. Serotype Ia clustered in CC23 and serotype V in CC1, while Ib strains clustered into CC10 (75.0%), CC17 (16.7%), and CC23 (8.3%). Sepsis observed in 10 (55.6%) EOD cases and 18 (69.2%) LOD cases were caused by CC17, while meningitis in 9 (56.3%) EOD cases and 23 (69.7%) LOD cases were caused by CC17. Invasive serotype III strains were significantly associated with ST17 and CC17 (*p* < 0.001). CC17 was not only detected as serotype III (57 of strain ST17 and 1 of strain ST188), but also found as serotype Ib/ST357 (2 strains).

### Hypervirulent GBS adhesion gene

The *hvgA* gene was detected in 66.7% of the invasive GBS isolates (62/93, Table 2), and 95% of CC17 isolates (57 of 60) carried the *hvgA* gene. Additionally, the *hvgA* gene was also found in CC10 (3), CC19 (1), and CC23 (1). All *hvgA* genes were detected in the serotype III isolates (61 of 74 serotype III isolates, 82.4%), except one serotype Ib/CC17 isolate. The isolates carrying *hvgA* caused infections mainly manifesting as meningitis (32, 65.3%) and sepsis (30, 68.2%).

### Antimicrobial resistance phenotypes and genotypes

Universal antimicrobial susceptibility to β-lactam antibiotics (penicillin and ampicillin) was detected, while low levels of resistances were observed for levofloxacin (8.6%), ofloxacin (2.2%), and azithromycin (1.1%). 93.5% of isolates (87) were resistant to tetracycline, owing to the *tetO* gene in 74.7% (65/87) and *tetM* gene in 46.0% (40/87) of GBS isolates, while the *tetL and tetK* genes were not observed (Table 3). Moreover, both *tetO* and *tetM* genes were detected in 2 of the 6 tetracycline-susceptible isolates. Erythromycin resistance (60.2%) was mostly due to modifications of the ribosomes, linked to the presence of *ermB* (87.5%, 48 isolates), and in 3.6% (2 isolates) of cases to an efflux mechanism encoded by the genetic determinant *mefA.* The *ermA* gene was not found, but *ermB* was detected in 30.0% (6/20) of erythromycin-susceptible isolates. Resistance to clindamycin was observed in 61 isolates (65.6%) and there were another 12 isolates (12.9%) exhibiting erythromycin-induced clindamycin resistance. The *linB* gene was identified in 24.6% (15/61) of clindamycin-resistant isolates. The resistance to tetracycline, erythromycin, and clindamycin was not significantly different between serotypes or CC17 isolates, but resistance to clindamycin was higher in GBS isolates with *hvgA* than the isolates without (*p* < 0.05)*.*

**Table 3:** Comparison of resistance to tetracycline, erythromycin, and clindamycin of GBS isolates with different serotypes, CC17 and *hvgA*

	Total (*n* = 93)	Serotypes (n,%)				CCs (n,%)		*hvgA* (n,%)	
		Ia (*n* = 4)	Ib (*n* = 12)	III (*n* = 74)	IV (*n* = 3)	CC17 (*n* = 60)	Non-CC17 (*n* = 33)	*hvgA*(+) (*n* = 62)	*hvgA*(−) (*n* = 31)
Tetracycline	87 (93.5%)	4 (100%)	11 (91.7%)	69 (93.2%)	3 (100%)	57 (95.0%)	30 (90.9%)	60 (96.8%)	27 (87.1%)
Erythromycin	56 (60.2%)	2 (50%)	7 (58.3%)	45 (60.8%)	2 (66.7%)	38 (63.3%)	18 (54.5%)	39 (62.9%)	17 (54.8%)
Clindamycin	61 (65.6%)	1 (25%)	8 (66.7%)	50 (67.6%)	2 (66.7%)	40 (66.7%)	21 (63.6%)	46 (74.2%)^a^	15 (48.4%)

^a^Compared to the *hvgA*(+) isolates, *P* < 0.05

## Discussion

GBS remains a leading cause of infant sepsis and meningitis worldwide within the first 90 days of life. The recent meta-analysis by Madrid et al. showed the incidence of invasive GBS disease in infants was 0.49 per 1000 live births worldwide, with serotype III (61.5%) being the dominant type and with 97% of cases caused by just five serotypes (Ia, Ib, II, III, and V) [4]. In the present study, serotypes Ia, Ib, III, and V were detected, and serotype III was the dominant serotype observed (79.6%), which is higher than the global average of 61.5% cited above, but similar to estimates from France [9], Italy [20] and other parts of China [7, 13].GBS vaccines covering five serotypes (Ia, Ib, II, III and V) have already entered Phase III clinical trials [16]; an approved vaccine would cover 95% of global newborn GBS infection [2, 5, 21], and would protect from all serotypes detected in this study. Additionally, in contrast to the approximately 50% of GBS-related meningitis cases caused by serotype III in infants worldwide [21, 22], serotype III accounted for 62.5 and 81.8% in EOD and LOD cases with meningitis in this study group, respectively.

In the present study, we obtained 15 STs from 93 invasive GBS isolates, of which 61.3% were caused by the highly pathogenic genotype ST17. The five types of CCs detected in this study (CC17, CC1, CC10, CC19, and CC23) are also the most common clones globally. Among 93 isolates, more than half of neonatal GBS infection were caused by the highly pathogenic strain CC17 (ST17, ST357, ST188). We found serotype III was more commonly associated with the most virulent clone, clonal complex (CC17). CC17 accounted for 78.4% of serotype III strains, and mainly caused the observed meningitis. Studies have shown that less than 12% of GBS strains with ST17 colonize adults, whereas CC17, in contrast, presents as highly pathogenic and virulent among both infants with under-developing immune systems and immunocompromised patients [10]. In the process of occurrence and development of neonatal invasive infection, CC17 strains enhance the pathogenicity and invasiveness of GBS through a special virulence mechanism, which is the main cause of meningitis in these patients (> 80%) [9, 10, 23, 24]. CC17 colonization is the main risk factor for neonatal GBS infection [25, 26]. A study from the Netherlands found that the main contributing factor to the increase in incidence of GBS infection between 1987 and 2011 was the increase in prevalence of CC17 [25]. Manning SD et al. [23] found ST17 and ST19 were the local dominating genotypes of neonatal invasive infections in Canada, and that ST1, ST12, and ST23 were the primary genotypes among pregnant women. In China, similar to the results of that study, ST17 was found as the main genotype for newborns infected with GBS, and ST19 for pregnant women [27]. CC17 and CC19 are the most common important gene pedigrees of GBS serotype III, and likely for that reason serotype III is the most common serotype causing GBS invasive disease in infants.

Our findings showed around 95% of serotype-III/CC17 isolates harbouring the *hvgA* gene, which suggested that *hvgA* is involved in the pathogenesis of invasive GBS infectious diseases. The ST17 strain without the selective *hvgA* gene presents low adhesion to the host cell, which is comparable to that of the non-ST17 strain [10]. The *hvgA* gene was previously known to be carried only by ST17, while our study found the *hvgA* gene was mainly detected from strains with CC17 (92%,57/62), while CC10, CC19, and CC23 strains also carried the *hvgA* gene. Moreover, a strain with serotype-Ib/ST357/CC17 had the *hvgA* gene as well. Our findings also indicated that most of the *hvgA* genes have been detected in serotype-III/ST17 strains, which is consistent with other reported findings [18, 28].

Antimicrobial susceptibility testing of GBS isolates is critical for selecting appropriate antibiotic treatment. We found all isolates were susceptible to β-lactam antibiotics over the study period. Some studies have found that there was a decrease in the susceptibility of GBS to β-lactam (such as penicillin and cephalosporin) [29–31]. This study found that 93.5% of GBS isolates showed resistance to tetracycline, comparable to France (90, 95.5%) [24, 26], Italy (93.3%) [20], and Beijing (100%) [13]. The rate of erythromycin resistance was 60.2%, which is higher than previous results from France (16.7, 13.8%) [24, 26], Italy (12%) [20], and the United States (32%) [2]. This high resistance to macrolide antibiotics including erythromycin and clindamycin is possibly due to the overuse of antibiotics in animal husbandry and humans in China in the past decades. We found 85.7% of the erythromycin-resistant strains carried the resistance gene *ermB*. In this study, tetracycline resistance was mainly mediated by *tetO* and *tetM*, with carrier gene rates of 75 and 46%, respectively, whereas European countries were mainly dominated by *tetM*, with gene rates of 95% in France [24, 26] and 97.1% in Italy [20]. Furthermore, it was observed that a small number of antibiotic-sensitive GBS strains carried resistant genes, such as tetracycline-sensitive strains and erythromycin-sensitive strains. We also found *linB* is an important reason for clindamycin resistance; among 61 strains of clindamycin-resistant strains, 15 (24.6%) strains had the *linB* gene. This is lower than observations in South Africa where 38% (11/29) of strains had *linB* [32]. In contrast, *linB* was not detected in some investigations from Korean [33] and Iran [34]. Our group’s previous work revealed multi-drug resistance clusters (tetracycline, aminoglycoside, macrolide/lincomycin, and others) in GBS CC17 hypervirulent isolates by using genomic analysis [35]. This suggests that the antibiotics recommended by the intrapartum antibiotic prophylaxis program to prevent neonatal GBS in southern China are in need of updating. On the other hand, in addition to strengthening the rational use of antibiotics, and taking into account the higher proportion of CC17 of neonatal infection at the local level, we suggest more attention should be put on GBS multi-drug resistance control.

Until now, many studies have reported on the resistance spectrum of GBS strains among colonized pregnant women, but by contrast, fewer studies have been performed on the resistance spectrum of neonatal GBS infection [2, 13, 20, 24, 26]. In this study, genotypes exhibiting resistance to tetracycline and erythromycin were significantly correlated with a drug-resistant phenotype. However, antibiotic resistance was not limited to a specific serotype or sequence type. There was no correlation between GBS resistance and serotype, gene sequence type, and type of disease presentation. A study by Poyart C. et al. demonstrated similar results [24], but other studies have found the opposite. Joubrel, C. et al. reported that GBS serotype had a correlation with resistance, with the serotype-III strain having a low resistance rate and a high resistance rate to serotype-V strain [26]. One possible reason might be due to differences in the study population.

Our study has several limitations. GBS isolates were only from one province in China, Guangdong, and the sample size is relatively small. Therefore, these findings may not uniformly represent the whole China. We also did not collect data of population-based incidence rate and maternal information, which prevented limited further population-level analysis.

## Conclusions

The molecular epidemiologic characteristics of GBS neonatal invasive infections in southern China were mainly comprised of serotype-III/CC17. In Guangdong, resistance to tetracycline was mainly associated with *tetO* and *tetM* genes, erythromycin resistance was associated with the presence of *ermB,* and clindamycin resistance was associated with *linB.* It is recommended to establish corresponding prevention and control measures accordingly. Further large scale studies and routine GBS surveillance are required to learn more about this infection and to prevent it from occurring.

## Data Availability

The datasets used and/or analysed during the current study are available from the corresponding author on reasonable request.

## Abbreviations

CCs: Clonal complexes
CI: Confidence intervals
CLSI: Clinical and Laboratory Standards Institute
CPS: Capsular polysaccharide
EOD: Early onset disease
GBS: Group B Streptococcus
*HvgA*: Hypervirulent GBS Hdhesin
LOD: Late onset disease
MLST: Multi-locus sequence typing
STs: Sequence types
WHO: World Health Organization

## References

[1] Libster R, Edwards KM, Levent F, Edwards MS, Rench MA, Castagnini LA, Cooper T, Sparks RC, Baker CJ, Shah PE (2012). Long-term outcomes of group B streptococcal meningitis. Pediatrics.

[2] Phares CR, Lynfield R, Farley MM, Mohle-Boetani J, Harrison LH, Petit S, Craig AS, Schaffner W, Zansky SM, Gershman K (2008). Epidemiology of invasive group B streptococcal disease in the United States, 1999-2005. JAMA.

[3] Tibussek D, Sinclair A, Yau I, Teatero S, Fittipaldi N, Richardson SE, Mayatepek E, Jahn P, Askalan R (2015). Late-onset group B streptococcal meningitis has cerebrovascular complications. J Pediatr.

[4] Madrid L, Seale AC, Kohli-Lynch M, Edmond KM, Lawn JE, Heath PT, Madhi SA, Baker CJ, Bartlett L, Cutland C (2017). Infant group B streptococcal disease incidence and serotypes worldwide: systematic review and meta-analyses. Clin Infect Dis.

[5] Johri AK, Lata H, Yadav P, Dua M, Yang Y, Xu X, Homma A, Barocchi MA, Bottomley MJ, Saul A (2013). Epidemiology of group B Streptococcus in developing countries. Vaccine.

[6] Verani JR, McGee L, Schrag SJ, Division of Bacterial Diseases NCfI, Respiratory Diseases CfDC, Prevention (2010). Prevention of perinatal group B streptococcal disease--revised guidelines from CDC, 2010. MMWR Recomm Rep.

[7] Guan X, Mu X, Ji W, Yuan C, He P, Zhang L, Huang Y, Li J, Chen J, Zhong H (2018). Epidemiology of invasive group B streptococcal disease in infants from urban area of South China, 2011-2014. BMC Infect Dis.

[8] Jones N, Bohnsack JF, Takahashi S, Oliver KA, Chan M-S, Kunst F, Glaser P, Rusniok C, Crook DWM, Harding RM (2003). Multilocus sequence typing system for group B streptococcus. J Clin Microbiol.

[9] Tazi A, Disson O, Bellais S, Bouaboud A, Dmytruk N, Dramsi S, Mistou M-Y, Khun H, Mechler C, Tardieux I (2010). The surface protein *HvgA* mediates group B streptococcus hypervirulence and meningeal tropism in neonates. J Exp Med.

[10] Tazi A, Bellais S, Tardieux I, Dramsi S, Trieu-Cuot P, Poyart C (2012). Group B Streptococcus surface proteins as major determinants for meningeal tropism. Curr Opin Microbiol.

[11] Ji W, Zhang L, Guo Z, Xie S, Yang W, Chen J, Wang J, Cheng Z, Wang X, Zhu X (2017). Colonization prevalence and antibiotic susceptibility of group B Streptococcus in pregnant women over a 6-year period in Dongguan, China. PLoS One.

[12] Lu B, Li D, Cui Y, Sui W, Huang L, Lu X (2014). Epidemiology of group B Streptococcus isolated from pregnant women in Beijing, China. Clin Microbiol Infect.

[13] Wang P, Tong J-j, Ma X-h, Song F-l, Fan L, Guo C-m, Shi W, Yu S-j, Yao K-h, Yang Y-h (2015). Serotypes, antibiotic susceptibilities, and multi-locus sequence type profiles of Streptococcus agalactiae isolates circulating in Beijing, China. PLoS One.

[14] Dangor Z, Cutland CL, Izu A, Kwatra G, Trenor S, Lala SG, Madhi SA (2016). Temporal changes in invasive group B Streptococcus serotypes: implications for vaccine development. PLoS One.

[15] Heath PT, Balfour G, Weisner AM, Efstratiou A, Lamagni TL, Tighe H, O'Connell LAF, Cafferkey M, Verlander NQ, Nicoll A (2004). Group B streptococcal disease in UK and Irish infants younger than 90 days. Lancet (London, England).

[16] Kobayashi M, Schrag SJ, Alderson MR, Madhi SA, Baker CJ, Sobanjo-Ter Meulen A, Kaslow DC, Smith PG, Moorthy VS, Vekemans J. WHO consultation on group B Streptococcus vaccine development: report from a meeting held on 27–28 April 2016. Vaccine. 2016. 10.1016/j.vaccine.2016.12.029. 10.1016/j.vaccine.2016.12.029 PMC6892266 28017431

[17] Simonsen KA, Anderson-Berry AL, Delair SF, Davies HD (2014). Early-onset neonatal sepsis. Clin Microbiol Rev.

[18] Teatero S, McGeer A, Low DE, Li A, Demczuk W, Martin I, Fittipaldi N (2014). Characterization of invasive group B streptococcus strains from the greater Toronto area, Canada. J Clin Microbiol.

[19] Gygax SE, Schuyler JA, Kimmel LE, Trama JP, Mordechai E, Adelson ME (2006). Erythromycin and clindamycin resistance in group B streptococcal clinical isolates. Antimicrob Agents Chemother.

[20] Imperi M, Gherardi G, Berardi A, Baldassarri L, Pataracchia M, Dicuonzo G, Orefici G, Creti R (2011). Invasive neonatal GBS infections from an area-based surveillance study in Italy. Clin Microbiol Infect.

[21] Edmond KM, Kortsalioudaki C, Scott S, Schrag SJ, Zaidi AKM, Cousens S, Heath PT (2012). Group B streptococcal disease in infants aged younger than 3 months: systematic review and meta-analysis. Lancet.

[22] Le Doare K, Heath PT (2013). An overview of global GBS epidemiology. Vaccine.

[23] Manning SD, Springman AC, Lehotzky E, Lewis MA, Whittam TS, Davies HD (2009). Multilocus sequence types associated with neonatal group B streptococcal sepsis and meningitis in Canada. J Clin Microbiol.

[24] Poyart C, Réglier-Poupet H, Tazi A, Billoët A, Dmytruk N, Bidet P, Bingen E, Raymond J, Trieu-Cuot P (2008). Invasive group B streptococcal infections in infants, France. Emerg Infect Dis.

[25] Bekker V, Bijlsma MW, van de Beek D, Kuijpers TW, van der Ende A (2014). Incidence of invasive group B streptococcal disease and pathogen genotype distribution in newborn babies in the Netherlands over 25 years: a nationwide surveillance study. Lancet Infect Dis.

[26] Joubrel C, Tazi A, Six A, Dmytruk N, Touak G, Bidet P, Raymond J, Trieu Cuot P, Fouet A, Kernéis S (2015). Group B streptococcus neonatal invasive infections, France 2007-2012. Clin Microbiol Infect.

[27] Lu B, Wang D, Zhou H, Zhu F, Li D, Zhang S, Shi Y, Cui Y, Huang L, Wu H (2015). Distribution of pilus islands and alpha-like protein genes of group B Streptococcus colonized in pregnant women in Beijing, China. Eur J Clin Microbiol Infect Dis.

[28] Bellais S, Six A, Fouet A, Longo M, Dmytruk N, Glaser P, Trieu-Cuot P, Poyart C (2012). Capsular switching in group B Streptococcus CC17 hypervirulent clone: a future challenge for polysaccharide vaccine development. J Infect Dis.

[29] Chu YW, Tse C, Tsang GKL, So DKS, Fung JTL, Lo JYC (2007). Invasive group B Streptococcus isolates showing reduced susceptibility to penicillin in Hong Kong. J Antimicrob Chemother.

[30] Dahesh S, Hensler ME, Van Sorge NM, Gertz RE, Schrag S, Nizet V, Beall BW (2008). Point mutation in the group B streptococcal pbp2x gene conferring decreased susceptibility to beta-lactam antibiotics. Antimicrob Agents Chemother.

[31] Morikawa Y, Kitazato M, Katsukawa C, Tamaru A (2003). Prevalence of cefotaxime resistance in group B streptococcus isolates from Osaka, Japan. J Infect Chemother.

[32] Bolukaoto JY, Monyama CM, Chukwu MO, Lekala SM, Nchabeleng M, Maloba MR, Mavenyengwa RT, Lebelo SL, Monokoane ST, Tshepuwane C (2015). Antibiotic resistance of Streptococcus agalactiae isolated from pregnant women in Garankuwa, South Africa. BMC Res Notes.

[33] Ryu H, Park YJ, Kim YK, Chang J, Yu JK (2014). Dominance of clonal complex 10 among the levofloxacin-resistant Streptococcus agalactiae isolated from bacteremic patients in a Korean hospital. J Infect Chemother.

[34] Mousavi SM, Nasaj M, Hosseini SM, Arabestani MR (2016). Survey of strain distribution and antibiotic resistance pattern of group B streptococci (*Streptococcus agalactiae*) isolated from clinical specimens. GMS Hyg Infect Control.

[35] Campisi E, Rosini R, Ji W, Guidotti S, Rojas-López M, Geng G, Deng Q, Zhong H, Wang W, Liu H (2016). Genomic analysis reveals multi-drug resistance clusters in group B Streptococcus CC17 hypervirulent isolates causing neonatal invasive disease in southern mainland China. Front Microbiol.

